# Outcome Prediction at Patient Level Derived from Pre-Treatment 18F-FDG PET Due to Machine Learning in Metastatic Melanoma Treated with Anti-PD1 Treatment

**DOI:** 10.3390/diagnostics12020388

**Published:** 2022-02-02

**Authors:** Anthime Flaus, Vincent Habouzit, Nicolas de Leiris, Jean-Philippe Vuillez, Marie-Thérèse Leccia, Mathilde Simonson, Jean-Luc Perrot, Florent Cachin, Nathalie Prevot

**Affiliations:** 1 Nuclear Medecine Department, Saint-Etienne University Hospital, University of Saint-Etienne, 42000 Saint-Etienne, France; vincent.habouzit@gmail.com (V.H.); nathalie.prevot@chu-st-etienne.fr (N.P.); 2 Nuclear Medicine Department, Hospices Civils de Lyon, University of Lyon, 69008 Lyon, France; 3 Nuclear Medicine Department, CHU Grenoble Alpes, University Grenoble Alpes, 38000 Grenoble, France; ndeleiris@chu-grenoble.fr (N.d.L.); jpvuillez@chu-grenoble.fr (J.-P.V.); 4 Laboratoire Radiopharmaceutiques Biocliniques, University Grenoble Alpes, INSERM, CHU Grenoble Alpes, 38000 Grenoble, France; 5 Dermatology Department, CHU Grenoble Alpes, University Grenoble Alpes, 38000 Grenoble, France; mtleccia@chu-grenoble.fr; 6 Jean Perrin Cancer Centre of Clermont-Ferrand, Nuclear Medicine Department, 63011 Clermont-Ferrand, France; mathildes@startmail.com (M.S.); florent.cachin@clermont.unicancer.fr (F.C.); 7 Dermatology Department, Saint-Etienne University Hospital, University of Saint-Etienne, 42000 Saint-Etienne, France; j.luc.perrot@chu-st-etienne.fr; 8 INSERM U 1059 Sainbiose, University of Saint-Etienne, 42000 Saint-Etienne, France

**Keywords:** 18F-FDG PET, radiomics, machine-learning, immunotherapy, melanoma, prediction

## Abstract

(1) Background: As outcome of patients with metastatic melanoma treated with anti-PD1 immunotherapy can vary in success, predictors are needed. We aimed to predict at the patients’ levels, overall survival (OS) and progression-free survival (PFS) after one year of immunotherapy, based on their pre-treatment 18F-FDG PET; (2) Methods: Fifty-six metastatic melanoma patients—without prior systemic treatment—were retrospectively included. Forty-five 18F-FDG PET-based radiomic features were computed and the top five features associated with the patient’s outcome were selected. The analyzed machine learning classifiers were random forest (RF), neural network, naive Bayes, logistic regression and support vector machine. The receiver operating characteristic curve was used to compare model performances, which were validated by cross-validation; (3) Results: The RF model obtained the best performance after validation to predict OS and PFS and presented AUC, sensitivities and specificities (IC95%) of 0.87 ± 0.1, 0.79 ± 0.11 and 0.95 ± 0.06 for OS and 0.9 ± 0.07, 0.88 ± 0.09 and 0.91 ± 0.08 for PFS, respectively. (4) Conclusion: A RF classifier, based on pretreatment 18F-FDG PET radiomic features may be useful for predicting the survival status for melanoma patients, after one year of a first line systemic treatment by immunotherapy.

## 1. Introduction

Immunotherapy using immune checkpoint inhibitors (ICI) that target programmed cell death 1 receptor (anti-PD1) has substantially changed the therapeutic strategies for cancers such as metastatic melanoma [1]. However, as only subsets of patients will take advantage from it, it is necessary to develop non-invasive tools to stratify pre-treatment risk and assess prognosis, in order to prevent toxicities and hasten the introduction of more appropriate treatments. Nevertheless, no tools are currently clinically identified as helpful to select patients and which tools to use remains an open question [2]. Moreover, traditional cohort-oriented methods (such as the Kaplan–Meier survival techniques [3] to investigate real-world evidence data) have shown limited results because of their difficulties to cope with heterogeneous patient populations and their inability to provide patient-level predictions [4,5].

Computational medical imaging—known as radiomics—involves the analysis and translation of medical images into quantitative data. It allows an in-depth characterization of tumor imaging phenotypes with the underlying hypothesis that images reflect not only the tissues’ macroscopic but also their cellular and molecular properties. These image driven biomarkers could serve as tools to better aid clinical decisions [6].

Glucose analog, 2-deoxy-2-[^18^F]fluoro-D-glucose (18F-FDG) uptake represents glucose metabolic activity and is used as a tracer of positron emission tomography (PET)/X-ray computed tomography (CT) for initial staging in cases of high-risk melanoma, macroscopic lymph node involvement and/or known distant metastases [7]. 

Few studies have examined the prognostic value of 18F-FDG PET radiomic features for metastatic melanoma before immunotherapy treatment, but associations have been found between 18F-FDG PET parameters and outcome at a group level [8,9,10]. However, to our knowledge, no study has previously investigated patient-level prediction performances before treating them with anti-PD1 as a first line therapy. Therefore, our aim was to assess the prediction performance of a pre-treatment 18F-FDG PET-based machine learning model for separating individual patients with metastatic melanoma, according to overall survival (OS) and progression-free survival (PFS), after one year of a first line systemic treatment, by immunotherapy (anti-PD1)

## 2. Materials and Methods

The protocol was approved (2 July 2020) by the institutional medical ethics committee (IRB: IORG0007394) of Saint-Etienne (IRBN 842020/CHUSTE) and all methods were carried out in accordance with relevant guidelines and regulations. Informed consent to participate in the study was obtained from all the patients according to national regulations.

### 2.1. Patients

The hospital information system of two university hospitals (Saint-Etienne and Grenoble) was investigated to identify metastatic melanoma patients treated by a first line of anti-PD1 treatment and imaged with an 18F-FDG PET-CT scan before therapy, between January 2016 and January 2019. Electronic clinical and radiological databases were used to obtain patients’ demographical details, clinical history and metastatic status—as defined by the eighth edition American Joint Committee on Cancer (AJCC) melanoma staging system [11], anatomopathological data, treatment data, clinical outcome and follow-up duration as well as 18F-FDG PET data.

Out of 64 screened patients, 56 were included. Inclusion criteria were as follows: (1) metastatic melanoma proven by biopsy; (2) anti-PD1 antibodies were the first line of treatment, with no previous systematic therapy; (3) patients were B-RAF wild type; and (4) pre-treatment 18F-FDG PET data were available. Eight patients were excluded: (1) patients with no measurable disease or no significant FDG avid tumor (*n* = 6), including patients presenting only brain metastases due to the impossibility of clearly delineating their metabolic tumoral volume; and (2) patients with lesion sizes inferior to 64 voxels (*n* = 2), since the radiomic analysis becomes irrelevant below this threshold [12,13].

### 2.2. Follow-Up of Patients 

Overall survival, progression-free survival or time to last censoring were recorded starting from the date of the PET scan. OS was defined as the time (in months) between the PET scan and the date of death, due to any cause. PFS was defined as the time between the PET scan and locoregional or distant relapse, or death from any cause. Living patients were censored at the time of the last clinical follow-up. Patient response was assessed with Response Evaluation Criteria in Solid Tumors (RECIST 1.1) using diagnostic contrast-enhanced CT and all available clinical information. 

### 2.3. Imaging Protocols

Scans were acquired using different PET-CT systems: Biograph mCT Flow 20, Biograph 6 HI-REZ and Biograph Horizon 16 (Siemens Healthcare, Erlangen, Germany) and Discovery 690 (General Electrics Healthcare, Chicago, IL, USA). Patients fasted for at least 6 h and blood glucose levels were confirmed to be under 180 mg/dl. Patients were injected according to current guidelines with an activity of 2.5–4 MBq/kg ^18^F-FDG (median activity 269 MBq, range 146–468 MBq). Sixty minutes after injection, whole-body PET and unenhanced CT images were acquired. The CT attenuation-corrected acquired data were reconstructed using a three-dimensional ordered subset expectation–maximization algorithm as previously described [10]. Each scanner used consistent reconstruction settings, matrix and voxel size. Each voxel in PET images were converted into standard uptake value (SUV) [14].

### 2.4. Radiomics: Feature Extraction 

A feature extraction process was performed using the free software LIFEx (v4.0, Local Image Feature Extraction) which is compliant with the image biomarker standardization initiative (IBSI) [13,15]. One experienced nuclear medicine physician analyzed and segmented 18F-FDG PET scans without knowledge of the patients’ clinical outcome. A semi-automatic method was employed for segmentation based on a 40% SUVmax threshold [16]. Each hypermetabolic lesion was segmented to create a volume of interest (VOI) that was equivalent to the metabolic tumoral volume (MTV).

For each patient, radiomic features were extracted from the VOI of the lesion with the highest 18F-FDG uptake. This was consistent with the Positron Emission Tomography Response Criteria in Solid Tumors (PERCIST) definition of target lesion [17]. A total of 45 features were computed for each VOI after voxel intensity resampling using 64 discrete values between 0 and 32 SUV units [18].

The 14 first order features were: 4 features from shape, 5 from histogram and 5 from conventional indexes (SUVmin, SUVmean, SUVmax, SUVpeak and total lesion glycolysis or TLG). The 31 second order textural features (derived from different matrices) were: 6 from the grey level co-occurrence matrix (GLCM), 11 from the grey-level run length matrix (GLRLM), 3 from the neighborhood grey-level different matrix (NGLDM) and 11 from the grey-level zone length matrix (GLZLM). A detailed description of each parameter is available in the technical appendix of the LIFEx software [13].

### 2.5. Radiomics: Feature Harmonization 

Since different PET scanners were used, post-reconstruction harmonization method was performed for all PET parameters using the ComBat harmonization method in R with a software package (https://github.com/Jfortin1/ComBatHarmonization (accessed on 5 August 2020). ComBat harmonization was described in 2007 [19] and is now widely used in genomics: its efficacy in PET has been shown [20].

### 2.6. Radiomics: Feature Selection 

A feature reduction procedure is necessary to select a subset of useful features that increase the prediction accuracy and reduce overfitting [21]. First, multicollinearity of the features were analyzed by Spearman correlation, with a threshold for the correlation coefficient of 0.8 [22]. Secondly, information gain ratio [23], Gini index [21] and F-score [24] were used as ranking and filter-based feature selection methods to reduce feature dimension. Radiomic features were ranked based on the three selection methods according to their correlation with survival status at 1-year follow-up. A total of 5 best-ranked features were selected based on the overall ranks across the 3 scoring methods [25]. Selected features were standardized using the standard scaler function to unit variance within the training set and the same scaler was used to transform the test set. Synthetic minority oversampling technique was used to correct for imbalanced datasets [26].

### 2.7. Machine Learning Approach for Classification

Five different machine learning algorithms used as classification models were compared, including neural network, logistic regression, support vector machine, random forest and naïve bayes. Given that the difference between our defined binary response and predicted response by each classification model can be described by a confusion matrix, we can define the following properties: the number of true positives (TP), true negatives (TN), false positives (FP) and false negatives (FN). The models’ performances were then evaluated by calculating the area under the curve (AUC) of the receiver operating characteristic (ROC) curve and mapping the models’ sensitivity and specificity measured by the TPR and 1–TNR, respectively [27]. Each model’s performance was validated using a sampling technique method: “cross-validation”. The patients’ data were randomly separated into training sets and testing sets stratified on the outcome (training set size: 75%). The whole procedure was repeated 50 times and results were averaged.

The statistical analyses were performed using the open-source *R* software package [28] (version 3.0.1, http://www.Rproject.org (accessed on 1 August 2020). A Mann–Whitney U test or chi-square test enabled us to appropriately assess the difference between two quantitative variables or compare categorical data. The machine learning approach was performed using Python (version 3.7) and the open source Numpy, Scipy and Scikit-learn packages. All *p* values are two-sided, and a *p* value < 0.05 is considered statistically significant. 

## 3. Results

### 3.1. Patient Characteristics

A total of 56 patients were included with a median age of 68 years old (range: 40–84). The median follow-up was 22.1 months (range: 2.1–49.2). At one year, 28 patients (50%) presented progressive diseases and 16 patients (29%) had died. We found no statistically significant differences for any parameters—sex, age, university hospital, metastatic status, before starting anti-PD1 treatment, histological characteristics of the initial melanoma, localization or initial cancer staging—between patients alive or dead at one year, patients with progressive disease versus partial or complete response or patients with stable disease.

### 3.2. Selection of Radiomic Features for Prediction 

We ranked selected radiomic features according to the scoring methods, as shown in Table 1. For OS, the top five predictive parameters were: GLZLM long-zone emphasis, GLZLM long-zone high grey-level emphasis, GLZLM zone length non-uniformity, GLCM homogeneity and histogram kurtosis. For PFS, they were: GLZLM long-zone emphasis, GLZLM long-zone low grey-level emphasis, GLZLM long-zone high grey-level emphasis CONVENTIONAL SUV standard deviation and GLCM entropy log10. Parameters extracted from GLZLM were the most informative to predict OS and PFS in our cohort. 

### 3.3. Performances of the Five Machine Learning Methods for Patient Classification

The overall classification performances of the five models for OS and PFS in the validation set are shown in Table 2. The random forest model obtained the best performance for both OS and PFS. AUC (95% CI), sensitivities (95% CI) and specificities (95% CI) were 0.87 ± 0.1, 0.79 ± 0.11 and 0.95 ± 0.06 for OS and 0.9 ± 0.07, 0.88 ± 0.09 and 0.91 ± 0.08 for PFS, respectively.

## 4. Discussion

This study intended to predict survival status using pre-therapeutic 18F-FDG PET of metastatic melanoma patients, after one year of a first line systemic treatment by immunotherapy (anti-PD1). Five radiomic features, extracted from 18F-FDG PET, which captured information about tumor phenotype, were used to develop and validate five machine learning models. Using a cross validation method, we found the random forest classifier model predicted PFS and OS at one year with the highest accuracy. 

In this study, the five selected radiomics features were second order features, which possessed stronger correlations with outcome in this cohort than conventional 18F-FDG-PET metrics. Indeed, conventional metrics, i.e., SUVmax, SUVmean and MTV, were initially included in the extracted radiomics set but were not selected for analysis due to their lower contributions to the outcome prediction. The choice of the best radiomics parameters from the pretherapeutic 18F-FDG-PET to predict the outcome is still a matter of debate as results from previous studies are heterogeneous. One study reporting on SUVmax found SUVmean and tumor heterogeneity index were not correlated with OS or PFS in a heterogeneous cohort of 55 melanoma patients before anti-PD1 treatment [9]; however, other studies showed significant correlations between conventional or second order 18F-FDG PET parameters and melanoma patients’ outcomes after immunotherapy [8,10,29]. Authors showed conventional parameters, such as SUV max, SUVpeak, TLG and MTV, were associated with OS [8,10,29]. Second order features, such as tumor heterogeneity index, as well as long-zone emphasis from the GLZLM matrix were associated with OS [8,10]. Moreover, six second order features correlated with treatment response, among whom three came from the GLZLM matrix [29]. This is coherent with our ranking of radiomic features to predict PFS, which is best with three GLZLM features.

In line with previous studies, the model employing the random forest classifier—known as a classifier with high ability to learn and predict in small cohorts [30]—obtained the best performance among the machine learning classifiers in our cohort. In other studies, this classifier, based on radiomics, predicted disease recurrence accurately for non-small cell lung cancer [25], enabled risk stratification of gastro-intestinal stromal tumors [31] and helped differentiate recurrent brain tumors from radiation necrosis [32]. 

Previous studies have shown at group levels that 18F-FDG-PET may help discriminate melanoma patients that will benefit from immunotherapy versus patients with poor prognosis. The most informative parameters are total metabolic tumoral volume, bone to liver uptake ratio and two radiomics features: tumor heterogeneity index and GLZLM long-zone emphasis [8,9,10]. However, even if this information is of great importance, we can still refine our patient-level risk stratification. Our results using a classifier provide an additional biomarker. Similar results are available in non-small cell lung cancer [33].

Nevertheless, our study had some limitations. Firstly, its retrospective design and the descriptive nature of a small number of cases may limit it generalization to other cases. Secondly, as group level analyses suggested, clinical parameters were not significantly associated with outcome, so we only used pure radiomic modeling and did not integrate clinical parameters for further analysis and comparison [9]. Thirdly, we could not consider immune RECIST (iRECIST) as an endpoint for PFS since it would have introduced a chronological bias. Indeed, the RECIST working group developed iRECIST criteria in 2017 [34], while our data were gathered starting from January 2016. Recent studies have however demonstrated a relative agreement between RECIST and iRECIST [35] and no case of pseudo-progression was reported in our cohort. Fourthly, the use of different scanners may have affected the results of the 18F-FDG PET-based radiomic analyses [36]. However, in our work, intensity binning was performed on all data to increase the adherence to key methodological principles of radiomics analysis. Moreover, we conducted post-reconstruction harmonization using the ComBat method [20] to correct the effect of multi PET scanner as performed in other recent studies; this also underlined the feasibility of multicentric radiomics studies, which are scarce [37,38]. Finally, we did not examine the pathological correlations of these radiomic features, which are largely unknown and remain to be clarified in the future. 

## 5. Conclusions

To conclude, a random forest classifier based on 18F-FDG PET radiomic features may be useful to predict the survival status of metastatic melanoma patients after one year of treatment with immunotherapy (anti-PD1) as the first line choice. Such a biomarker presents the potential to help predict outcomes at the patient level and to improve risk stratification and treatment planning. A multicentric, prospective study, including more patients, should be performed to confirm the predictive performance of this biomarker. Following this first validation, a clinical trial with personalized treatment according to the 18F-FDG PET biomarker should be investigated. 

## Figures and Tables

**Table 1:** Ranking of the selected radiomic features extracted from the 18F-FDG PET, according to association with survival at one year.

Overall Survival			Progression Free Survival	
Overall Rank	Radiomic Feature		Overall Rank	Radiomic Feature
1	GLZLM_LZE		1	GLZLM_LZE
2	GLZLM_LZHGE		2	GLZLM_LZLGE
3	GLZLM_ZLNU		3	GLZLM_LZHGE
4	GLCM_Homogeneity		4	CONVENTIONAL_SUVstd
5	HISTO_Kurtosis		5	GLCM_Entropy_log10
6	GLRLM_GLNU		6	NGLDM_Coarseness
7	GLRLM_LRHGE		7	HISTO_Skewness
8	NGLDM_Coarseness		8	CONVENTIONAL_TLG
9	HISTO_Energy		9	HISTO_Entropy_log10
10	GLRLM_SRE		10	GLCM_Energy
11	HISTO_Entropy_log10		11	CONVENTIONAL_SUVmax
12	NGLDM_Contrast		12	GLRLM_GLNU
13	GLCM_Correlation		13	GLRLM_LRHGE
14	GLRLM_LGRE			
15	GLZLM_SZE			
16	HISTO_Skewness			
17	GLCM_Contrast			
18	CONVENTIONAL_SUVmin			

NGLDM: neighborhood grey-level different matrix, GLZLM: grey-level zone length matrix, GLRLM: grey-level run length matrix, HISTO: histogram, LZHGE: long-zone high grey-level emphasis; LZE: long-zone emphasis; LZLGE: long-zone low grey-level emphasis RLNU: run length non-uniformity, ZLNU: zone length non-uniformity; TLG: total lesion glycolysis, GLNU: grey-level non-uniformity, LRHGE: long-run high grey-level emphasis, GLCM: grey-level co-occurrence matrix, SZE: short-zone emphasis, SUV: standard uptake value, std: standard deviation.

**Table 2:** Performance of each model using cross-validation to predict overall survival and progression-free survival at one year, in patients with metastatic melanoma.

	Overall survival				
	NB	LR	RF	SVM	NN
AUC (95% CI)	0.82 ± 0.15	0.84 ± 0.15	0.87 ± 0.1	0.82 ± 0.15	0.84 ± 0.14
Sensitivity (95% CI)	0.78 ± 0.11	0.81 ± 0.11	0.79 ± 0.11	0.77 ± 0.12	0.79 ± 0.11
Specificity (95% CI)	0.86 ± 0.1	0.87 ± 0.10	0.95 ± 0.06	0.87 ± 0.09	0.89 ± 0.09
	Progression-free survival				
	NB	LR	RF	SVM	NN
AUC (95% CI)	0.69 ± 0.15	0.64 ± 0.14	0.90 ± 0.07	0.63 ± 0.15	0.69 ± 0.13
Sensitivity (95% CI)	0.80 ± 0.11	0.59 ± 0.14	0.88 ± 0.09	0.55 ± 0.14	0.58 ± 0.14
Specificity (95% CI)	0.57 ± 0.14	0.70 ± 0.12	0.91 ± 0.08	0.72 ± 0.12	0.81 ± 0.11

RF: random forest, LR: logistic regression, NN: neural network, SVM: support vector classification, NB: naïve bayes.

## Data Availability

The datasets used and/or analyzed during the current study are available from the corresponding author upon reasonable request. The data are not publicly available due to ethical restriction.
